# Heterografting with nonself rootstocks induces genes involved in stress responses at the graft interface when compared with autografted controls

**DOI:** 10.1093/jxb/eru145

**Published:** 2014-04-01

**Authors:** S. J. Cookson, M. J. Clemente Moreno, C. Hevin, L. Z. Nyamba Mendome, S. Delrot, N. Magnin, C. Trossat-Magnin, N. Ollat

**Affiliations:** ^1^EGFV, ISVV, INRA, UMR 1287, F-33140 Villenave d’Ornon, France; ^2^EGFV, ISVV, University of Bordeaux, UMR 1287, F-33140 Villenave d’Ornon, France; ^3^Santé et Agro-écologie du Vignoble, ISVV, INRA, UMR 1065 Villenave d’Ornon, France

**Keywords:** Gene expression, graft interface, grafting, grapevine, rootstock, stress response.

## Abstract

Although grafting is widely used in the agriculture of fruit-bearing crops, little is known about graft union formation in particular when two different species are grafted together. It is fascinating that two different plant species brought together can develop harmoniously as one organism for many decades. The objective of this study was to determine whether grafting two different grapevine genotypes alters gene expression at the graft interface in comparison to the presumably wound-like gene expression changes induced in autografts. Gene expression at the graft interface was studied 3, 7, 14, and 28 d after grafting in hetero- and autografts of grapevine (*Vitis* spp.). Genes differentially expressed between the hetero- and autografts during graft union formation were identified. These genes were clustered according to their expression profile over the time course. MapMan and Gene Ontology enrichment analysis revealed the coordinated upregulation of genes from numerous functional categories related to stress responses in the hetero- compared to the autografts. This indicates that heterografting with nonself rootstocks upregulates stress responses at the graft interface, potentially suggesting that the cells of the graft interface can detect the presence of a nonself grafting partner.

## Summary

Grafting together two different genotypes results in the upregulation of stress responses at the graft interface during graft union formation in comparison to the wound-like responses of autografts.

## Introduction

Grafting has been used in horticulture in China since before 2000 bc (Liu, 2006; Mudge *et al.*, 2009). Grafting is still widely used today, for example in the cultivation of grapevine, apples, *Prunus* spp., and vegetables (e.g. Lee *et al.*, 2010; Gregory *et al.*, 2013). In viticulture, grafting began in Europe to facilitate grapevine growth in soils infected with phylloxera, a soil-dwelling insect pest introduced to Europe from America (May, 1994). The role of rootstocks in defence responses may go beyond resistance to soil-borne pests and diseases: for example, in apple, certain rootstocks can confer resistance to fireblight to the scion (Jensen *et al.*, 2012). It has been suggested recently that grafting *per se* could increase plant defence responses by activating systemic defence mechanisms (Guan *et al.*, 2012). Despite the old and wise use of grafting in agriculture, the early stages of the grafting process and the molecular mechanisms involved in the communication between two different genotypes at the graft interface are still poorly understood.

Successful grafting is a complex process that begins with adhesion of the two grafted partners, followed by callus formation and the establishment of a functional vascular system (as reviewed by Pina and Errea, 2005). When callus cells come into contact, the cell walls undergo dissolution, holes in the cell walls appear, plasma membranes contact, and plasmodesmata appear (Jeffree and Yeoman, 1983).

The changes in graft union morphology and vascular formation have been studied using classical histology and various imaging techniques (e.g. Weatherhead and Barnett, 1986; Soumelidou *et al.*, 1994; Olmstead *et al.*, 2006; Leszczynski *et al.*, 2000; Bahar *et al.*, 2010; Milien *et al.*, 2012). At the molecular level, graft union formation presumably requires considerable reprogramming of gene expression, protein translation, and metabolism. Global changes in gene expression during the process of graft formation have been analysed in hickory (*Carya tomentosa*) with cDNA amplified fragment length polymorphism 0, 3, 7, and 14 d after grafting (Zheng *et al.*, 2010). This has revealed that some genes related to indole-3-acetic acid, cell cycle, metabolism, and signal transduction are differentially expressed (Zheng *et al.*, 2010). Graft union development in *Arabidopsis thaliana* hypocotyl grafts has been studied at the histological and transcriptional level (Yin *et al.*, 2012) and graft union development was shown to involve wound and hormone signalling and the clearing of cellular debris. However, hypocotyl grafting appears to be quite different from grafting in woody perennial species because the graft union formation is not accompanied by the development of callus tissue in the graft zone. In grapevine, grafting is traditionally performed on overwintering stems in the spring so that graft union formation and the reactivation of metabolism and growth of the cambium in the spring occur in parallel. In a previous study, this study group has shown that graft union development in grapevine involves the upregulation of many genes involved in cell-wall synthesis, wound responses, secondary metabolism, and signalling (Cookson *et al.*, 2013). These previous gene expression studies have identified genes involved in autografting of the same species (e.g. Zheng *et al.*, 2010; Yin *et al.*, 2012; Cookson *et al.*, 2013) but have not examined gene expression induced by grafting different species together.

To date, it is not known whether any degree of nonself recognition occurs between two different genotypes or species at a graft interface. Empirically it is known that not all species or genotypes graft together successfully and graft incompatibility has been widely reported (as reviewed by Pina and Errea, 2005). It has long been known that incompatible heterografts develop less mechanical strength and fewer trans-union xylem connections than compatible grafts (Yeoman and Brown, 1976; Yeoman *et al.*, 1978). However, the underlying causes of graft incompatibility are unknown and incompatible responses can arise many years after grafting has taken place. Incompatibility between different genotypes at the graft interface has been associated with the accumulation of phenolic compounds (Pina and Errea, 2008*b*), reactive oxygen species (Nocito *et al.*, 2010), and UDP-glucose pyrophosphorylase (Pina and Errea, 2008*a*), and modifications of cell-wall composition (Pina *et al.*, 2012).

In this work, gene expression was studied at the graft interface in hetero- and autografts of grapevine during a time course (3, 7, 14, and 28 d) after grafting using whole-genome microarrays. Many genes were differentially expressed over time, from 3 to 28 d after grafting, but they will not be discussed here as they have been described previously (Cookson *et al.*, 2013). The scion/rootstock graft interfaces studied were: the autograft *Vitis vinifera* cv. ‘Cabernet-Sauvignon N’ (CS; CS/CS) and the heterografts CS/*V. riparia* cv. ‘Riparia Gloire de Montpellier’ (RG; CS/RG) and CS/*V. berlandieri* x *V. rupestris* hybrid cv. ‘1103 Paulsen’ (1103P; CS/1103P). Many genes were differentially expressed between the different graft interface zones (i.e. between the graft interfaces made of only CS cells and those made up of cells of two different genotypes; data not shown). However, the present paper will focus on the identification of genes differentially regulated during the time course between the hetero- and autografted plants.

## Materials and methods

Two independent grafting experiments were done in the spring of 2011 and 2012. The samples from 2011 were used for microarray and quantitative real-time PCR (qPCR) analysis whereas the samples from 2012 were used only for qPCR analysis.

### Plant material and grafting procedure

Hardwood from CS (clone 15), RG (clone 1030), and 1103P (clone 198) was collected from a vineyard in France in January and stored as 1-m-long stems in a cold chamber (4 °C) until grafting in March. The scion CS was grafted onto RG (CS/RG) and 1103P (CS/1103P) as well as autografted onto CS rootstocks (CS/CS) (as described by Cookson *et al.*, 2013).

### RNA extraction

Three pools of 15 graft interface zones (approximately 5mm in length including both scion and rootstock tissues) were harvested 3, 7, 14, and 28 d after grafting for CS/RG, CS/1103P, and CS/CS and were immediately snap frozen in liquid nitrogen. Total RNA was extracted using the protocol described by Cookson *et al.* (2013).

### Microarray analysis

The microarray hybridizations were done for 36 samples (three pools of graft interface zones of CS/RG, CS/1103P, and CS/CS harvested 3, 7, 14, and 28 d after grafting) by the Plateforme Biopuces, Institut National des Sciences Appliquées, Toulouse, France according to the manufacturer’s instructions. The microarrays used were the grape whole-genome microarrays from Nimblegen, Roche (design name 090918 *Vitus* exp HX12) and were background corrected, quantile-normalized, and summarized as described by Cookson *et al.* (2013). The raw and normalized microarray data is available at http://www.ebi.ac.uk/arrayexpress (last accessed 20 March 2014) (accession number E-MTAB-1610).

The genes differentially expressed between the scion/rootstock combinations at 3–7, 7–14, and 14–28 d after grafting were identified using limma (Smyth, 2005; log_2_ fold-change >1, *P*<0.05, adjusted with Holm). This method identified genes that were differentially expressed between the hetero- and autografts between each time point of the time course: these genes were interesting because they behaved differently during graft union formation, depending of the scion/rootstock combination under consideration. The genes differentially expressed between CS/RG and CS/CS were further analysed using K-means clustering; the number of clusters chosen was selected from visual inspection of a hierarchical clustering dendrogram of the data (Supplementary Fig. S1 available at *JXB* online).

Differences in gene expression were visualized using MapMan (Thimm *et al.*, 2004; Usadel *et al.*, 2005). The MapMan mapping file was obtained from http://www.gomapman.org/ (last accessed 20 March 2014); 27 837 of the 29 549 genes on the microarray are present in the mapping file (Rotter *et al.*, 2009). Enrichments of functional categories of the MapMan annotation in the significantly differentially expressed genes were tested for significance by applying Fisher exact tests with a Bonferroni correction for multiple tests using Mefisto version 0.23beta (http://www.usadellab.org, last accessed 20 March 2014). Enrichment of Gene Ontology (GO) terms in significantly differentially expressed genes was evaluated using analysis tool from AgriGO (http://bioinfo.cau.edu.cn/agriGO, last accessed 20 March 2014; Du *et al.*, 2010) with Fisher tests and Bonferroni multiple testing correction (*P*<0.05).

### qPCR analysis

For qPCR experiments, genomic DNA contamination was removed from the RNA using a Turbo DNA-free kit (Ambion) and reverse transcription was done using the Superscript III kit (Invitrogen) using oligo dT primers and 1.5 μg RNA, both according to the manufacturer’s instructions. Gene expression was analysed on a Biorad CFX96 machine using iQ Sybr Green Supermix with a primer concentration of 250nM, according to the manufacturer’s instructions. The quality and quantity of cDNA synthesized was tested using two sets of primers that amplified the 3′ and 5′ regions of the same reference gene (a SAND protein, *VIT_06s0004g02820*, which was not differentially expressed between the samples) and genomic DNA contamination was checked by qPCR using intron-specific primers (Supplementary Table S1). The expression of genes of interest was normalized using *VIT_02s0025g01050* (an additional reference gene which was also not differentially expressed between the samples); this gene was also used to confirm the stability of expression of *VIT_06s0004g02820* (quantified using the 3′ primers, Supplementary Table S1). PCR efficiency for each primer pair was calculated using LinRegPCR (Ramakers *et al.*, 2003). Data are expressed as deltadeltaCq, with a CS/CS sample used as the reference.

## Results

### Heterografting with a nonself rootstock alters the expression of many genes at the graft interface in comparison to autografting

**Fig. 1.**
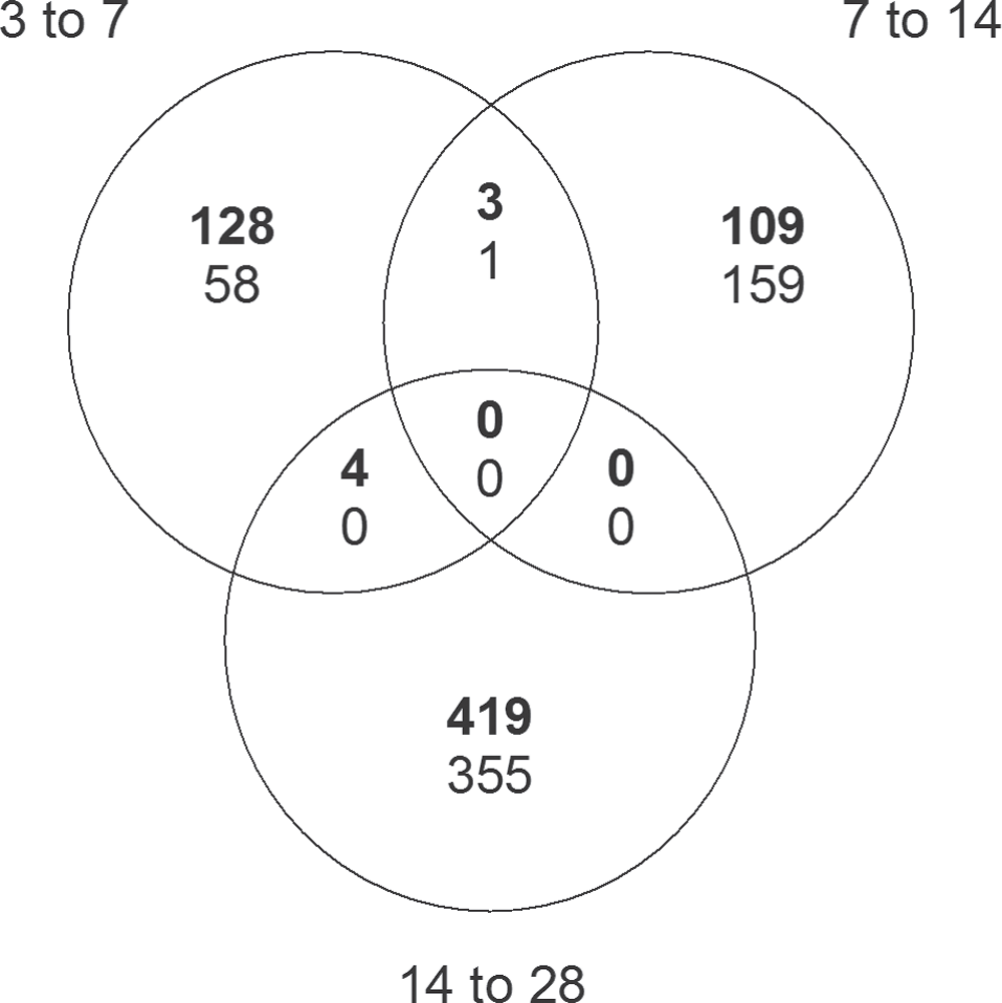
Transcriptomic analysis of the graft interface of the grapevine heterograft CS/RG and autograft CS/CS at 3, 7, 14, and 28 d after grafting, showing the number of genes differentially expressed after grafting (log_2_ fold-change >1, *P*<0.05, adjusted with Holm). Upper values indicate upregulation; lower values indicate downregulation.

The gene expression differences between the graft interface of the heterograft CS/RG and the autograft were analysed at 3–7, 7–14, and 14–28 d after grafting: a total of 1105 differentially expressed genes were identified (Fig. 1, Supplementary Table S2). These 1105 genes behaved differently during graft union formation between the auto- and heterografts. A similar result was obtained when the graft interfaces of CS/CS and CS/1103P were compared (Supplementary Fig. S2A) as there were very few genes differentially expressed at the graft interface between the two heterografted plants (Supplementary Fig. S2B). Because of the similarity between the transcriptional response of CS/RG and CS/1103P to grafting, only the results of the comparison between CS/RG and CS/CS are described in this manuscript; some results for the comparison between CS/1103P and CS/CS are given in Supplementary Fig. S2.

The MapMan categories (BINs; Thimm *et al.*, 2004; Usadel *et al.*, 2005) enriched in all the 1105 genes differentially expressed between CS/CS and CS/RG were associated with cell walls, development (storage proteins), jasmonate signalling (lipoxygenase), various miscellaneous enzymes, and not assigned genes (Table 1). Among these genes, the GO compartments membrane, external encapsulating structure, and extracellular region/space/part were enriched, along with the functions catalytic and oxidoreductase activity (Table 2).

**Table 1.** Enrichment of MapMan functional categories (BINs) in the 1105 genes differentially expressed in the callus 3, 7, 14, and 28 d after grafting between the heterograft CS/RG and the autograftContingency gives the number of genes (i) from the BIN in the input list, (ii) in the background (microarray), (iii) not in the BIN in input list, and (iv) not in the background. *P*-values adjusted with Bonferroni. NA, not assigned.

BIN code	BIN name	Contingency	Adjusted *P*-value
10	Cell wall	41–515–988–26 293	9.80E-03
33.1	Development, storage proteins	9–42–1020–26 766	2.84E-02
17	Hormone metabolism	43–540–986–26 268	5.77E-03
17.7	Hormone metabolism, jasmonate	11–29–1018–26 779	4.38E-05
17.7.1	Hormone metabolism, jasmonate, synthesis/degradation	11–28–1018–26 780	3.29E-05
17.7.1.2	Hormone metabolism, jasmonate, synthesis/degradation, lipoxygenase	7–9–1022–26 799	2.40E-04
26	Miscellaneous enzymes	131–1596–898–25 212	1.21E-12
26.4	Miscellaneous enzymes, β-1,3 glucan hydrolases	10–38–1019–26 770	2.52E-03
26.1	Miscellaneous enzymes, cytochrome P450	32–305–997–26 503	3.72E-04
26.3	Miscellaneous enzymes, gluco-, galacto-, and mannosidases	12–68–1017–26 740	1.10E-02
26.8	Miscellaneous enzymes, nitrilases, nitrile lyases, berberine bridge enzymes, reticuline oxidases, troponine reductases	13–79–1016–26 729	1.01E-02
35	NA	324–11 861–705–14 947	7.07E-14
35.3	NA, new	67–3837–962–22 971	4.06E-12
35.1.5	NA, no ontology, pentatricopeptide repeat-containing protein	5–558–1024–26 250	1.83E-02

**Table 2.** Enrichment of GO terms in the 1105 genes differentially expressed in the callus 3, 7, 14, and 28 d after grafting between the heterograft CS/RG and the autograftContingency gives the number of genes (i) in the BIN in the input list, (ii) in the background (microarray), (iii) not in the BIN in input list, and (iv) not in the background. *P*-values adjusted with Bonferroni.

GO accession number	Term type	Term	Contingency	Adjusted *P*-value
GO:0005576	C	Extracellular region	106–1139–672–17 499	2E-10
GO:0005615	C	Extracellular space	37–255–741–18 452	2E-07
GO:0044421	C	Extracellular region part	37–271–741–18 436	7E-07
GO:0030312	C	External encapsulating structure	43–468–735–18 233	4E-04
GO:0016020	C	Membrane	305–6073–473–12 366	0.026
GO:0003824	F	Catalytic activity	491–9610–287–8643	6E-06
GO:0016491	F	Oxidoreductase activity	130–1836–648–16 758	8E-06

Key: F, molecular function and C, cellular compartment.

### Clustering reveals the divergent profiles of the genes differentially expressed during the time course

**Fig. 2.**
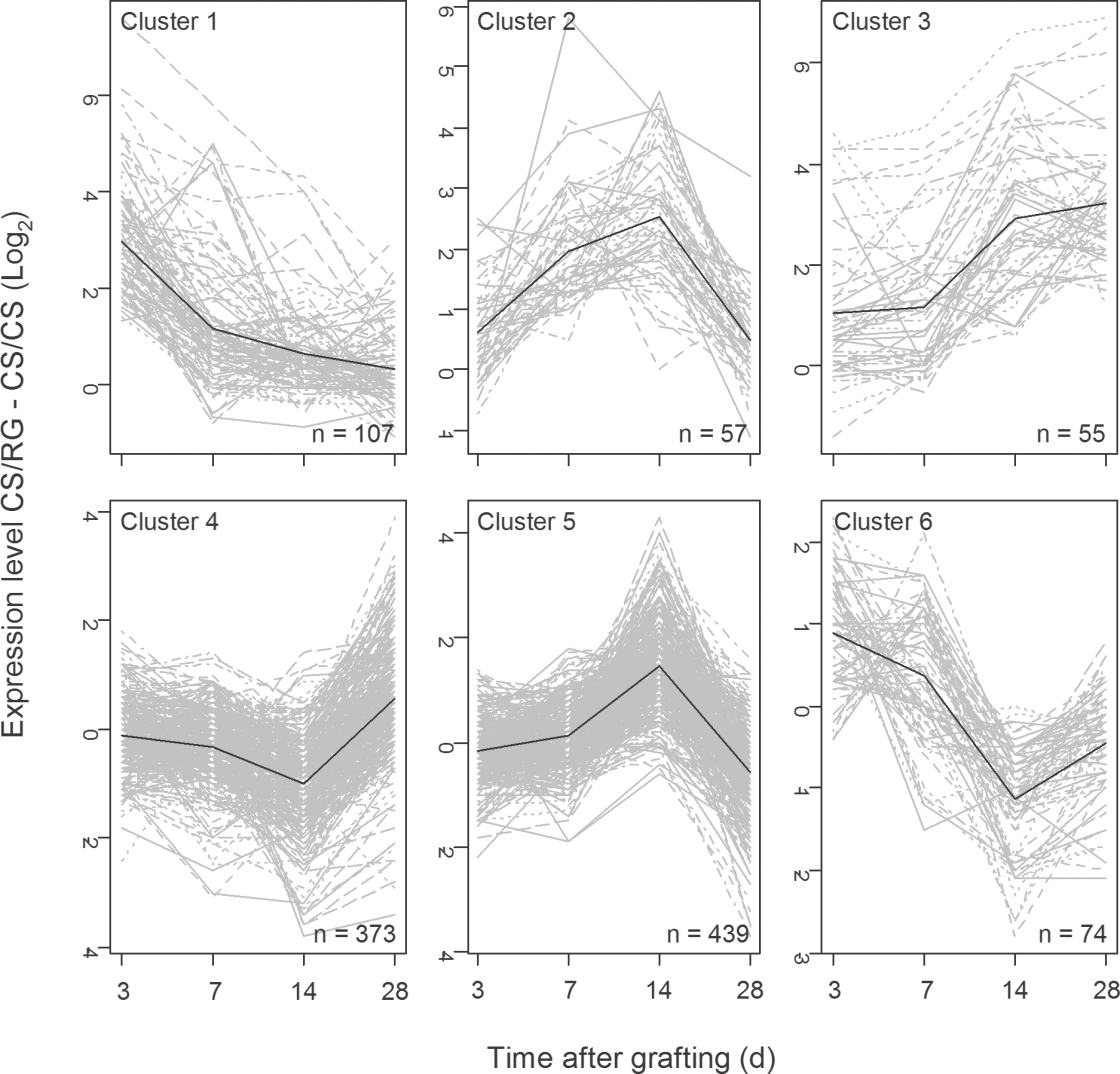
Clustering of gene expression profiles of genes differentially expressed between the heterograft CS/RG and the autograft control at the graft interface in the time course after grafting.

In order to simplify the interpretation of the genes differentially expressed between CS/CS and CS/RG, the profiles of gene expression were clustered into six clusters (Fig. 2, Supplementary Table S3). The expression of six of these genes was confirmed by qPCR (Supplementary Fig. S3).

Cluster 1 contained genes that were particularly upregulated in the heterograft CS/RG in comparison to the autograft control 3 d after grafting (Fig. 2). The functional categories were associated with cell wall, jasmonate signalling (lipoxygenase), miscellaneous enzymes, not assigned genes, and pathogenesis-related (PR) proteins were enriched in these early responding differentially expressed genes (Supplementary Table S4). Examination of the genes in cluster 1 identified the early upregulation in the heterograft of two dormancy-associated proteins (*VIT14s0083g00250* and *VIT_14s0083g00290*), some genes involved in oxidative stress (e.g. a peroxiredoxin *VIT_05s0020g00600*, a peroxidase *VIT_08s0058g00990*, l-ascorbate oxidase *VIT_06s0009g01320*), and PR protein 10 (*VIT_07s0005g00930*) (Supplementary Table S3).

Cluster 2 contained genes particularly upregulated 7 and 14 d after grafting in the heterograft CS/RG in comparison to the autograft control (Fig. 2). The functional category polyamine oxidase was enriched in this cluster (Supplementary Table S4 available at *JXB* online) along with the GO term catalytic activity (Supplementary Table S5). This cluster included two ankyrin repeat family proteins (*VIT_00s0256g00010* and *VIT_00s0256g00020*) which may be involved in protein–protein interactions, two genes from the category DNA (*VIT_08s0058g00090* and *VIT_16s0050g02320*), and two peroxidases (*VIT_12s0055g01010* and *VIT_12s0055g01030*) (Supplementary Table S3).

Cluster 3 contained genes that were particularly upregulated in the heterograft CS/RG towards the end of the time course, 14–28 d after grafting (Fig. 2). In this cluster, genes associated with secondary metabolism (particularly phenylpropanoid) were enriched (Supplementary Table S4). Cluster 3 contained four genes involved cell organization (*VIT_12s0059g00050*, *VIT_14s0081g00370*, *VIT_14s0081g00400*, and *VIT_14s0081g00420*), *SENESCENCE ASSOCIATED GENE 101* (*SAG101 VIT_05s0077g01730*), a lateral organ boundaries gene (*VIT_00s0340g00090*), and five receptor kinases (*VIT_04s0008g00920*, *VIT_17s0000g02360*, *VIT_12s0028g01430*, *VIT_15s0021g00840*, *VIT_15s0045g 00680*, and *VIT_18s0041g01770*) (Supplementary Table S3).

Cluster 4 contained genes upregulated 28 d after grafting in the heterograft CS/RG compared to autograft control (Fig. 2); the categories cell wall (particularly cellulose synthesis), miscellaneous enzymes, not assigned genes, secondary metabolism (flavonols), S-locus glycoprotein-like receptor kinases, and peptide/oligopeptide transport were enriched in this cluster (Supplementary Table S4). The GO compartment membrane and the functions kinase, molecular transducer, oxidoreductase, receptor, signal transducer, and transferase activities were enriched in this cluster (Supplementary Table S5). Cluster 4 also included a number of receptor kinases, two genes from the category DNA (*VIT_19s0014g05320* and *VIT_07s0130g00370*), and a number of transcription factors, including a SET domain-containing protein (*VIT_08s0056g01660*) (Supplementary Table S3).

Cluster 5 contained genes that were upregulated 14 d after grafting and then downregulated 28 d after grafting (Fig. 2); the functional categories β-1,3 glucan hydrolyases and trehalose-6-phosphate phosphatase were enriched in this cluster (Supplementary Table S4 available at *JXB* online) along with the GO terms catalytic activity and the extracellular compartment (Supplementary Table S5). In addition, cluster 4 contained two SAG101 genes (*VIT_05s0077g01720* and *VIT_14s0066g01830*), one SAG6 gene (*VIT_00s0301g00100*) (Supplementary Table S5), four PR proteins (*VIT_18s0001g03570*, *VIT_09s0002g06880*, *VIT_10s0003g03690*, and *VIT_12s0034g01230*), and many transcription factors, receptor kinases, and genes involved in calcium signalling (Supplementary Table S3).

Finally, cluster 6 contained genes that were strongly downregulated in the heterograft CS/RG compared to autograft control 14 d after grafting (Fig. 2). The categories development (storage proteins), jasmonate signalling, miscellaneous enzymes, and both abiotic and biotic (proteinase inhibitors) stress were enriched in this cluster (Supplementary Table S4) along with the GO term extracellular region (Supplementary Table S5). This cluster included six PR proteins including two tumour-related proteins (*VIT_17s0119g00150* and *VIT_17s0119g00230*) (Supplementary Table S5) and a number of transcription factors, including *GROWTH REGULTING FACTOR 3* (*VIT_09s0002g01350*) (Supplementary Table S3).

### Differential expression of genes associated with biotic stress

One of the common themes emerging from the genes differentially expressed between CS/CS and CS/RG during the time course is the differential expression of many genes involved in defence and/or stress responses. A summary of the gene expression differences of genes from the MapMan BIN biotic stress responses between the heterograft CS/RG and the autograft is given in a MapMan visualization (Supplementary Fig. S4 available at *JXB* online). The strong upregulation of genes from the functional categories jasmonate, cell wall, PR proteins, and secondary metabolites 3 d after grafting was evident (Supplementary Fig. S4A). Gradually, the expression of most genes associated with biotic stress decreased over time except the resistance genes (i.e. biotic stress receptors), which remained upregulated throughout the time course (Supplementary Fig. S4).

### A conceptual model of the gene expression changes at the graft interface during heterograft union formation

**Fig. 3.**
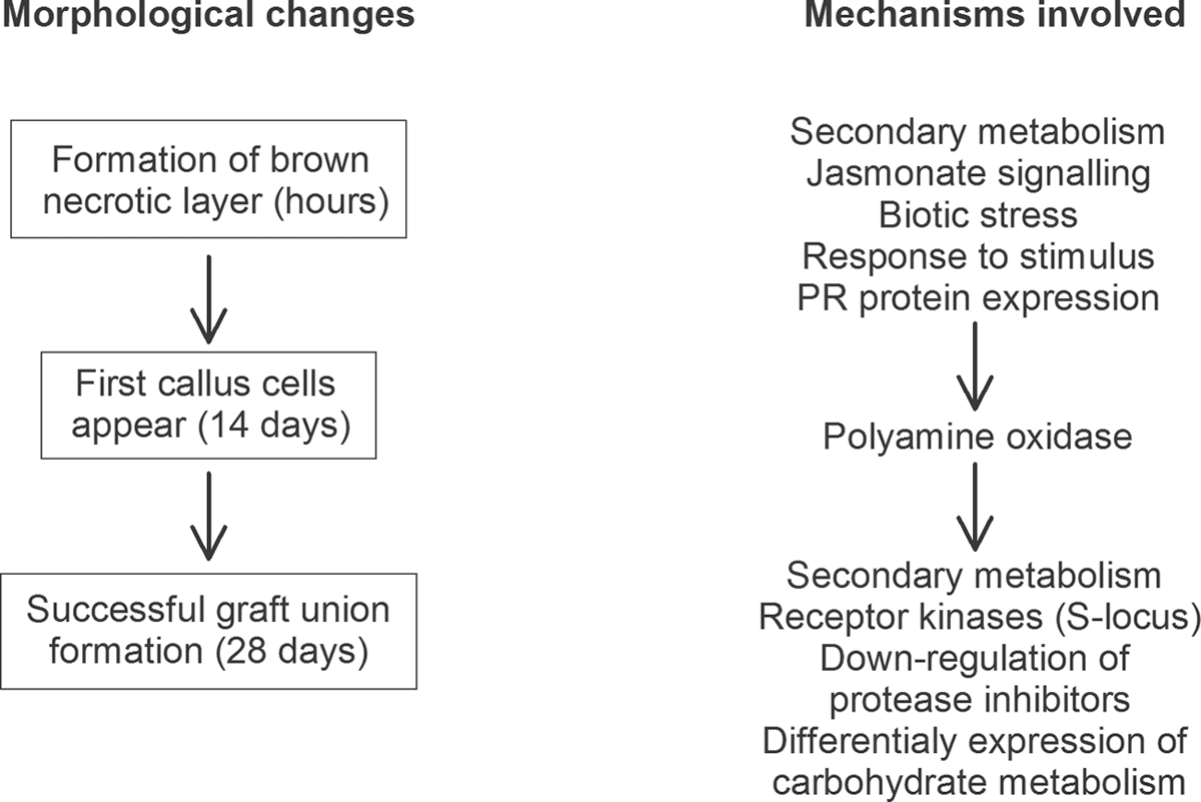
Conceptual model of the mechanisms involved in grafting together different plant genotypes.

In grapevine, graft union formation in both auto- and heterografts began (within a few hours) with the formation of a brown necrotic layer and the first callus cells developing 14 d after grafting. By 28 d after grafting, considerable callus tissue had developed and the graft union was functional (Fig. 3). Morphological changes are accompanied by many gene expression changes in autografted plants (Cookson *et al.*, 2013). Compared to autografting, heterografting resulted in additional gene expression changes: for example, the rapid upregulation of genes involved in jasmonate signalling, PR protein expression, and biotic stress and the sustained upregulation of many genes involved in secondary metabolism. Some polyamine oxidase genes were upregulated 7–14 d after grafting and S-locus receptor kinases were generally upregulated and protease inhibitors generally downregulated towards the end of graft union formation (14–28 d after grafting) (Fig. 3).

## Discussion

### Genes differentially expressed in a heterograft and autograft of grapevine

The objective of this work was to determine whether the transcriptome of grapevine is altered in response to grafting with a nonself genotype during the first month after grafting. As early as 1930, Kostoff suggested that the two different genotypes at the graft interface communicate with each other and induce an immune response (Kostoff, 1930). The presence of cell-wall projections on the cells of the graft interface suggested that cellular recognition processes are involved (Yeoman *et al.*, 1978; Jeffree and Yeoman, 1983). In addition, a difference in protein profile between *in vitro* callus auto- and heterografts of *Prunus* spp. has been reported (Pina and Errea, 2008*a*).

The gene expression differences at the graft interface between auto- and heterografts could be difficult to interpret because they may be related to a number of factors. In this study, the formation of the graft union concurred with the spring-time reactivation of metabolism in both auto- and heterografts; therefore, differences in gene expression between the different genotypes could be due to their different responses to warming in the spring. However, many genes were differentially expressed between the auto- and heterografts but not differentially 3–28 d after grafting (data not shown). Additionally, there could be differences in the timings of wound-healing processes in the different scion/rootstock combinations; however, in all combinations, the callus tissue was visible from 14 d after grafting and was well developed by 28 d after grafting (data not shown).

### Grafting with nonself rootstock triggers the differential expression of a large number of genes involved in plant defence and/or stress responses

Plant defence and/or stress responses are designed to facilitate plant survival under abiotic stresses and/or to hinder pathogen development and progression of the disease upon pathogen infection. There are many similarities in the recognition and signalling pathways of abiotic and biotic stress responses (e.g. as reviewed by Tippmann *et al.*, 2006); therefore, it is difficult to determine whether biotic or abiotic pathways were differentially regulated at the graft interface of hetero- and autografts.

Local pathogen attack can induce a form of programmed cell death, the hypersensitive response (as reviewed by Van Breusegem and Dat, 2006). Typically, the cells involved in the hypersensitive response generate an oxidative burst by producing reactive oxygen species, superoxide anions, hydrogen peroxide, hydroxyl radicals, and nitrous oxide. These responses are not unique to biotic stress responses and can also be induced in response to numerous abiotic stress conditions (as reviewed by Tippmann *et al.*, 2006). The differential expression of genes involved in oxidative stress in this work (e.g. glutathione S-transferases, ascorbate oxidase, polyphenol oxidases, and peroxidases) could be associated with the induction of an oxidative burst at the graft interface. The enrichment of polyamine metabolism in cluster 2 was due to the presence of polyamine oxidase in this cluster: polyamine oxidase has been shown to be a key element of oxidative burst to induce programmed cell death (Yoda *et al.*, 2006). Grafting in plants is presumably perceived as a considerable stress by the plant involved, and the grafting of two different genotypes described in this work further upregulates oxidative stress responses at the transcriptional level. Oxidative stress has also been implicated in the grafting incompatibility response of *in vitro* heterocallus grafts (Nocito *et al.*, 2010): the activity of five antioxidant enzymes was increased in incompatible pear/quince heterografts in comparison to the compatible pear/pear autografts.

Plant genomes contain large numbers of receptor kinases with very divergent extracellular domains and functions. The receptor kinases that were differentially expressed in response to grafting with nonself genotypes included genes from the functional categories LRR, kinase receptor-like cytoplasmic kinase VII, S-locus glycoprotein-like, DUF 26, and wheat LRK10-like. LRR proteins are the largest group of receptor kinases in plants and the motif is thought to be involved in signal transduction and to mediate protein–protein interactions. LLR domain-containing proteins have been implicated in many developmental pathways and defence responses (as reviewed by Haffani *et al.*, 2004). S-locus glycoprotein-like receptor kinases were first identified as being important in self-incompatibility responses in Brassica flowers and have since been shown to be involved in plant defence responses (as reviewed by Sanabria *et al.*, 2008). LRK10 receptor kinases were first identified as leaf rust resistance genes in wheat and are similar in structure to S-locus motifs. The biological functions of receptor-like cytoplasmic kinases are much less understood; it has been suggested that their lack of an apparent extracellular domain implies that receptor-like cytoplasmic kinases more likely function in signal transduction rather than ligand perception (Lu *et al.*, 2010). The differential expression of receptor kinases in hetero- compared to autografts could suggest that there is some degree of nonself recognition at the graft interface in heterografted plants.

Genes associated with jasmonate signalling also particularly enriched in the genes upregulated in the heterograft CS/RG compared to the autograft plants at early time points after grafting. Jasmonate and its derivates are involved in wound and defence signalling (as reviewed by Browse, 2009); therefore, the upregulation of genes involved in jasmonate signalling could also be associated with defence and wound responses during the early stages of heterografting.

Towards the end of the time course, 28 d after grafting, cell-wall precursor and lignin synthesis were enriched in the genes upregulated in the heterograft CS/RG compared to the autograft control: this could also be due to differences in plant wounding and defence responses. The upregulation of phenylalanine ammonia-lyase (PAL) gene expression has been reported during the formation of *in vitro* callus grafts of apricot and plum (Pina and Errea, 2008*b*). PAL expression was further increased by the callus grafting of incompatible heterograft (apricot/plum and plum/apricot) combinations in comparison to both autograft control samples (apricot/apricot and plum/plum) (Pina and Errea, 2008*b*). The upregulation of PAL expression was accompanied by the presence of soluble and wall-bound phenolic compounds but not by the production of lignin during the first 3 weeks after callus grafting (Pina and Errea, 2008*b*).

PR proteins form part of the general mechanism of plant responses to unfavourable conditions and are considered the executioners of plant immune responses as they generally have antifungal or bacterial properties (such as β-1–3 glucanase, thaumatin, chitinase, and defensins). PR proteins were particularly enriched in the genes upregulated between the autograft and the heterograft at early time points after grafting. This could suggest that the wound response to grafting induces PR proteins but that the induction is not maintained at later time points due to absence of pathogens. Upregulation of PR1 expression has also been observed in sterile *in vitro* callus grafts of pear/quince heterografts in comparison to pear/pear autograft control (Nocito *et al.*, 2010). However, upregulation of PR1 in heterografts could also be related to its function in hormone signalling (Naseem *et al.*, 2012).

It has recently been proposed that grafting with nonself genotypes confers an increase in plant defence responses by activating systemic defence mechanisms (Guan *et al.*, 2012); however, this may be difficult to demonstrate because of the indirect effects of rootstock on plant development: e.g. mineral nutrition, vigour). The work presented here shows a possible means by which grafting with nonself rootstocks could activate defence mechanisms, by the differential regulation of numerous defence-related genes at the graft interface of heterografted plants. Interestingly, gene expression is altered in the shoot apex of the autograft control compared with the heterograft CS/RG, but there are no gene expression differences between the heterografts CS/RG and CS/1103P (Cookson and Ollat, 2013). Furthermore, a number of defence-related genes are differentially expressed between CS/CS and CS/RG in the shoot apex (Cookson and Ollat, 2013), specifically the downregulation of secondary metabolism and PR protein expression and the differential regulation of a number of receptor kinases.

### Hybrid necrosis in grapevine suggests that CS and RG have some degree of immune incompatibility

The observation that hybrids between *V. vinifera* and *V. riparia* show high levels of hybrid necrosis (Filler *et al*., 1994*a*,*b*) could suggest that autoimmune-induced hybrid necrosis is involved. It has been suggested that hybrid necrosis is due to genetic incompatibilities that involve an immune response (Bomblies and Weigel, 2007). Wheat plants undergoing hybrid necrosis were found to have increased levels of superoxide (a molecule involved in oxidative burst and hypersensitive response) (Khanna-Chopra *et al.*, 1998). Elevated levels of PR proteins and defence response genes have also been associated with hybrid necrosis (Bomblies *et al.*, 2007). The genes that have been identified as responsible for hybrid necrosis to date have been involved in receptor kinase-mediated defence responses (as reviewed by Bomblies, 2009). The idea that immune responses are involved in grafting two different genotypes together and hybrid necrosis was proposed in 1930 (Kostoff, 1930) and has only relatively recently resurfaced in the literature (e.g. Wulff *et al.*, 2004; Bomblies and Weigel, 2007). The gene expression differences observed at the graft interface of heterografted grapevines known to induce hybrid necrosis (CS is *V. vinifera* and RG is *V. riparia*) adds further support to this hypothesis.

## Conclusion

Grafting with nonself genotypes triggers the differential expression of numerous genes at the graft interface during the first month after grafting; this begins with the upregulation of oxidative stress responses and PR proteins and is followed by the upregulation of many other genes involved in plant stress responses. These findings suggest that the cells at the graft interface are capable of detecting the presence of the nonself grafting partner, which may induce an immune-type response.

## Supplementary material

Supplementary data are available at *JXB* online.

Supplementary Table S1. Sequences and PCR efficiency of primers used for qPCR analysis.

Supplementary Table S2. Gene expression differences between the graft interface zones of the heterograft CS/RG and the autograft 3, 7, 14, and 28 d after grafting.

Supplementary Table S3. Clustering of genes differentially expressed between the heterograft CS/RG and the autograft in the time course after grafting.

Supplementary Table S4. Enrichment of MapMan BINs in the six clusters shown in Fig. 2.

Supplementary Table S5. Enrichment of GO terms in the six clusters shown on Fig. 2.

Supplementary Fig. S1. Cluster dendrogram of the expression profiles of genes differentially expressed between the heterograft CS/RG and the autograft in the time course after grafting.

Supplementary Fig. S2. Transcriptomic analysis of the graft interface 3, 7, 14, and 28 d after grafting in different scion/rootstock combinations of grapevine heterografts CS/RG and CS/1103P and the autograft.

Supplementary Fig. S3. Validation of microarray data by qPCR in the graft interface.

Supplementary Fig. S4. MapMan visualization of genes assigned to the functional category biotic stress differentially expressed between the heterograft CS/RG and the autograft control at 3, 7, 14, and 28 d after grafting.

Supplementary Data
