# Supplementary material for: Heterografting with nonself rootstocks induces genes involved in stress responses at the graft interface when compared with autografted controls
Source: J Exp Bot. 2014 Apr 1;65(9):2473–81. doi: 10.1093/jxb/eru145 (PMC4036518; doi:10.1093/jxb/eru145)
Supplement: Supplementary Data [file supp_65_9_2473__index.html]

Heterografting with nonself rootstocks induces genes involved in stress responses at the graft interface when compared with autografted controls — Heterografting with nonself rootstocks induces genes involved in stress responses at the graft interface when compared with autografted controls — Supplementary Data 

# Heterografting with nonself rootstocks induces genes involved in stress responses at the graft interface when compared with autografted controls

## Supplementary Data

Data files

**Files in this Data Supplement:**

- Supplementary Data - Supplementary Data
- Supplementary Data - Supplementary Data
- Supplementary Data - Supplementary Data
