# Supplementary material for: Heterografting with nonself rootstocks induces genes involved in stress responses at the graft interface when compared with autografted controls
Source: J Exp Bot. 2014 Apr 1;65(9):2473–81. doi: 10.1093/jxb/eru145 (PMC4036518; doi:10.1093/jxb/eru145)
Supplement: Supplementary Data [file supp_eru145_jexbot120600_file001.pdf]

Journal of Experimental Botany

Title: Hetero-grafting with non-self rootstocks induces genes involved in stress responses at the graft interface when compared with auto-grafted controls

Authors: Sarah Jane Cookson, Maria José Clemente Moreno, Cyril Hevin, Larissa Zita Nyamba Mendome, Serge Delrot, Noel Magnin, Claudine Trossat-Magnin, Nathalie Ollat

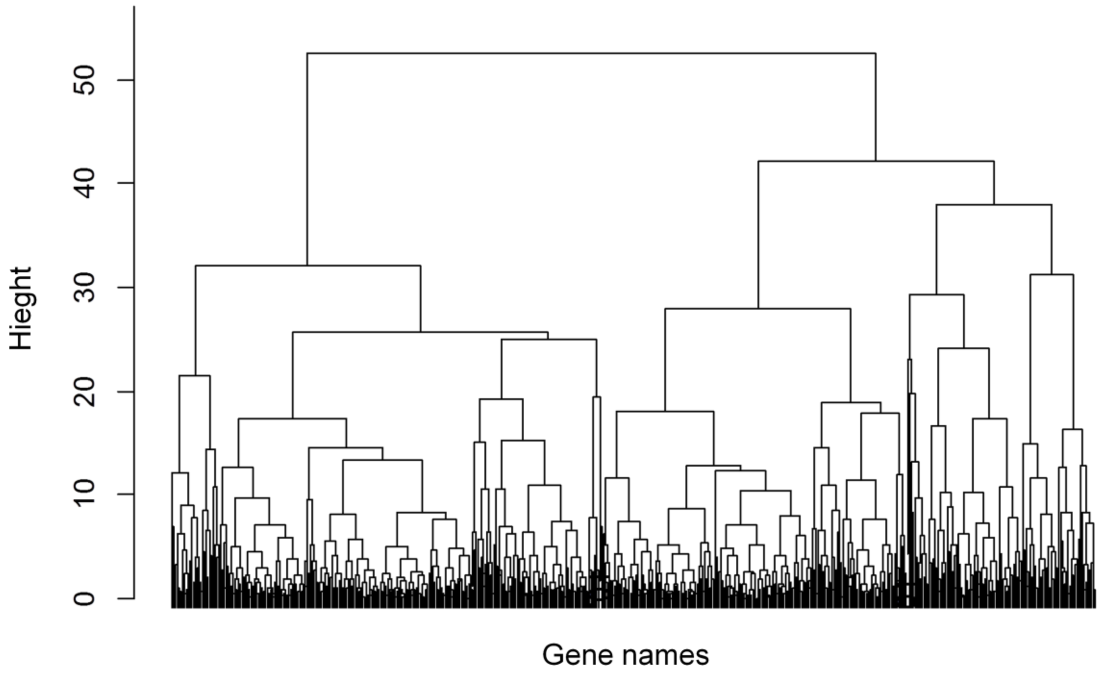


Figure S1 Cluster dendrogram of the gene expression profiles of the genes differentially expressed between CS/RG and CS/CS during the time course of gene expression changes after grafting.

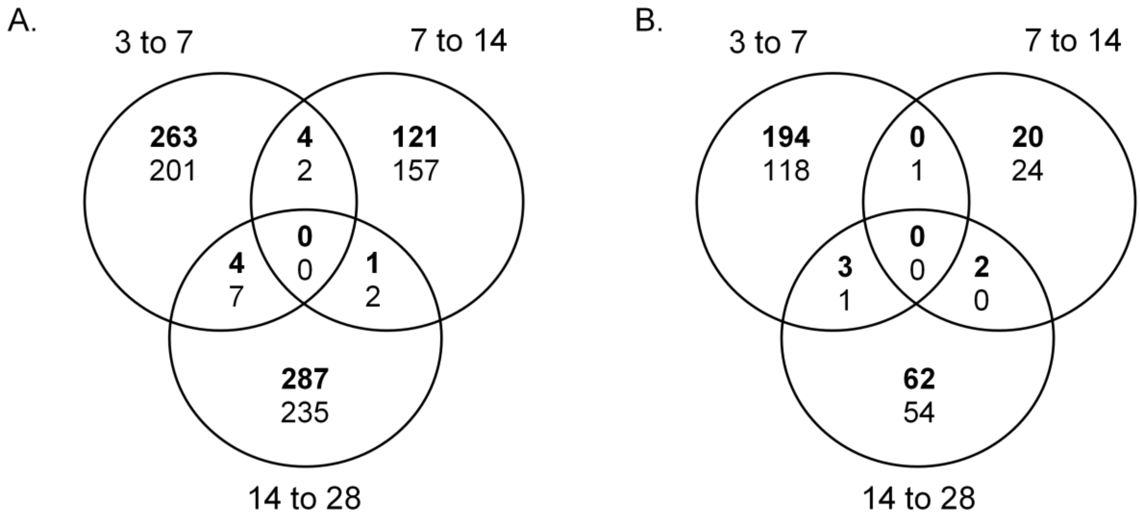


Figure S2. Transcriptomic analysis of the graft interface 3, 7, 14 and 28 d after grafting in different scion/rootstock combinations of grapevine A) hetero-grafts CS/1103P and auto-grafts CS/CS and B) hetero-grafts CS/1103P and CS/RG. Venn Diagrams of the number of genes differentially expressed between the different genotypes from 3 to 7, from 7 to 14 and from 14 to 28 d after grafting (up- and down-regulated genes shown in bold and normal text respectively, $\log_2$ fold change > 1, $p < 0.05$, adjusted with Holm).

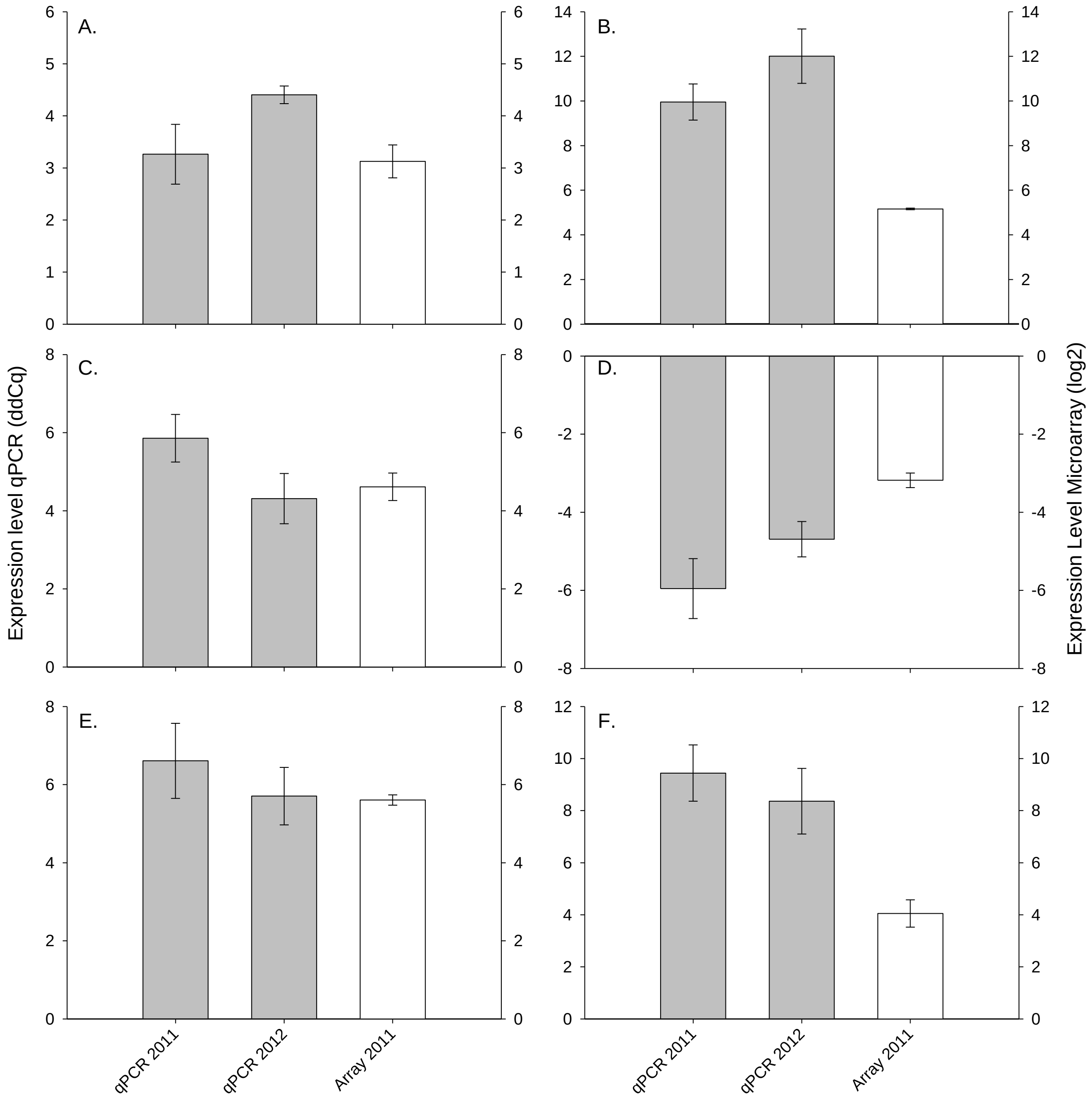


Figure S3. Validation of microarray data (open bars, samples from 2011) by qPCR (filled bars, samples from 2011 and 2012) in the graft interface A.) *VIT_04s0008g03990* 28 d after grafting, B.) *VIT_09s0002g01350* 14 d after grafting, C.) *VIT_05s0094g00280* 3 d after grafting, D.) *VIT_15s0045g00680* 28 d after grafting, E.) *VIT_16s0098g00480* 28 d after grafting and F.) *VIT_08s0040g02770* 14 d after grafting. Means and standard errors shown, n = 3.

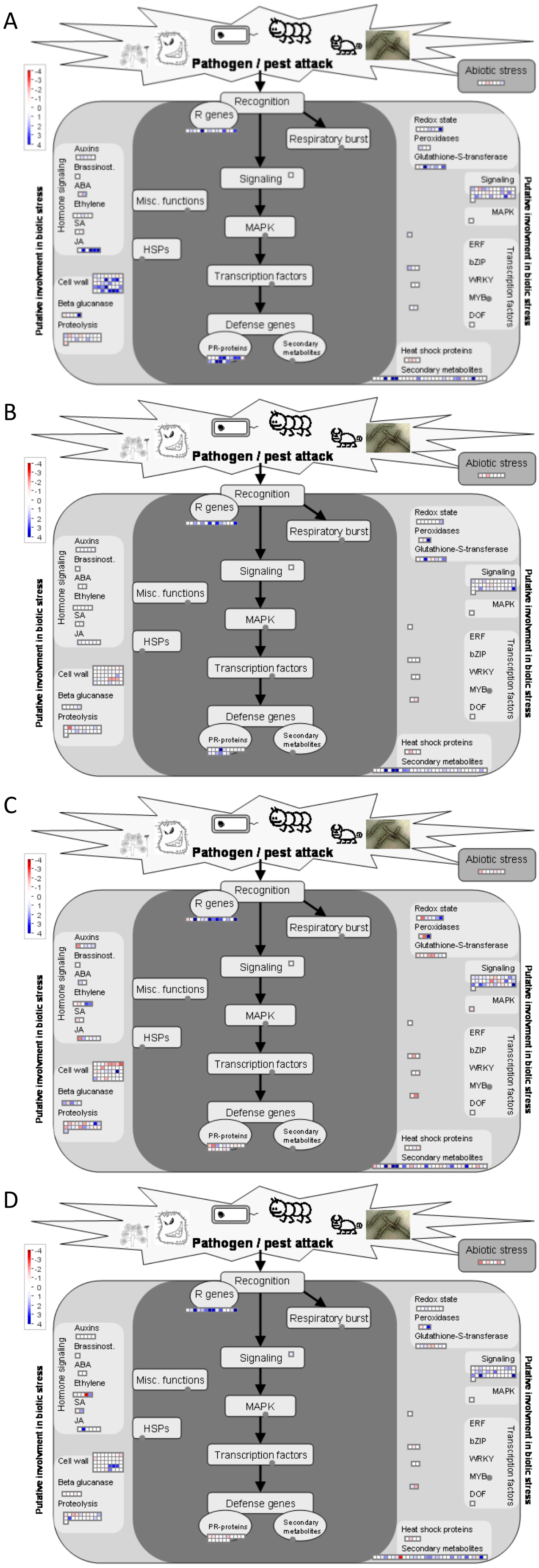
A
B
C
D
Pathogen / pest attack
Abiotic stress
Recognition
R genes
Respiratory burst
Signaling
Misc. functions
MAPK
HSPs
Transcription factors
Defense genes
PR-proteins
Secondary metabolites
Auxins
Brassinost.
ABA
Ethylene
SA
JA
Hormone signaling
Cell wall
Beta glucanase
Proteolysis
Putative involvment in biotic stress
Redox state
Peroxidases
Glutathione-S-transferase
ERF
bZIP
WRKY
MYB
DOF
Heat shock proteins
-4
-3
-2
-1
0
1
2
3
4

Figure S4. MapMan visualization of genes assigned to the functional category biotic stress, visualization of genes differentially expressed between the hetero- (CS/RG) and auto-graft (CS/CS) A) 3, B) 7, C) 14 and D) 28 d after grafting. Squares represent the expression of individual genes from each sub-category, the degree of up- and down- regulated gene expression is shown in shades of blue and red respectively.

Table S1. Sequence and mean PCR efficiency of primers used for qPCR analysis.

| **Gene code** | **Gene function** | **Use of primer** | **Orientation of primer** | **Primer sequences ('5-'3)** | **Mean PCR efficiency** |
|---|---|---|---|---|---|
| *VIT_06s0004g02820* | SAND protein | 5' test | Forward | ACCCCTTTGCTC GGAGGAACAGAT | 2.028 |
| | | | Reverse | ACCTGAAGCTTG CCTTGTCGCA | |
| *VIT_06s0004g02820* | SAND protein | 3' test & reference gene | Forward | TGCTGGGTTACC CCGGAGTTTGA | 1.967 |
| | | | Reverse | CAGACCCGGTTG CACGTCCG | |
| *VIT_06s0004g02820* | SAND protein | genomic DNA test | Forward | CTGTGCACCTGC TTCGCCCA | 1.954 |
| | | | Reverse | AGGAATGGGGA GAGGGGTCCT | |
| *VIT_02s0025g01050* | Ubiquitin conjugating enzyme E2-17 | Reference gene | Forward | TCCTCCTGACAG TCCATATGCTGG T | 1.87 |
| | | | Reverse | GGGCTGGGCTCC ACTGCTCC | |
| *VIT_04s0008g03990* | Cell wall degradation | Gene cluster 4 | Forward | CTGACACCCCCA CTGAAGAC | 1.92 |
| | | | Reverse | ACGGGGTAACAA AGTGGCTT | |
| *VIT_09s0002g01350* | Transcription factor | Gene cluster 6 | Forward | AGAGACTCCTGG CTTGACCT | 1.879 |
| | | | Reverse | AAGTACCAGTTT GGATTCTGGA | |
| *VIT_05s0094g00280* | Class IV chitinase | Gene cluster 1 | Forward | ACAATCCTGAAA CGGTGGCT | 1.886 |
| | | | Reverse | TGATGGCTCGAA TTGTGGCT | |
| *VIT_15s0045g00680* | Biotic stress receptor | Gene cluster 3 | Forward | AAGGCTCGAAAG GTTCCCTG | 1.962 |
| | | | Reverse | GGGTTTTGCTCT GCATTCCG | |
| *VIT_16s0098g00480* | Triacylglycerol lipase | Gene cluster 4 | Forward | CCACCTCTCTTG GGGAATGTC | 1.852 |
| | | | Reverse | ATTCAACAGTCC CTGCCTCAC | |
| *VIT_08s0040g02770* | Unknown | Gene cluster 2 | Forward | TGCCTTGGCACA AGACTCAT | 1.831 |
| | | | Reverse | GCAGTCCTCACT GGGTCAAA | |

Table S4. Enrichment of MapMan BINs in the 6 clusters shown on Figure 3. P values adjusted with Bonferroni.

| Cluster | Bin number | Bin name | p value | Adjusted p value |
|---|---|---|---|---|
| 1 | 10 | cell wall | 4.27E-06 | 3.29E-04 |
| | 17.7 | hormone metabolism, jasmonate | 3.80E-04 | 2.93E-02 |
| | 17.7.1 | hormone metabolism, jasmonate, synthesis/degradation | 3.53E-04 | 2.72E-02 |
| | 17.7.1.2 | hormone metabolism, jasmonate, synthesis/degradation, lipoxygenase | 2.29E-05 | 1.76E-03 |
| | 26 | miscellaneous enzymes | 4.78E-09 | 3.68E-07 |
| | 26.3 | miscellaneous enzymes, gluco-, galacto- and mannosidases | 1.08E-11 | 8.29E-10 |
| | 26.7 | miscellaneous enzymes, oxidases, copper, flavone etc. | 3.60E-04 | 2.77E-02 |
| | 35 | not assigned | 1.79E-07 | 1.38E-05 |
| | 35.3 | not assigned, new | 1.03E-04 | 7.95E-03 |
| | 16.1.5 | secondary metabolism, isoprenoids, terpenoids | 2.94E-05 | 2.26E-03 |
| | 20.1 | stress, biotic | 6.08E-04 | 4.68E-02 |
| | 20.1.7 | stress, biotic, PR-proteins | 1.18E-05 | 9.09E-04 |
| 2 | 22.2 | polyamine metabolism, degradation | 5.72E-05 | 3.89E-03 |
| | 22.2.1 | polyamine metabolism, degradation, polyamine oxidase | 5.72E-05 | 3.89E-03 |
| 3 | 16 | secondary metabolism | 1.49E-04 | 7.77E-03 |
| | 16.2 | secondary metabolism, phenylpropanoids | 3.16E-04 | 1.64E-02 |
| 4 | 10.2 | cell wall, cellulose synthesis | 6.91E-05 | 1.19E-02 |
| | 10.2.1 | cell wall, cellulose synthesis, cellulose synthase | 1.95E-05 | 3.38E-03 |
| | 26 | miscellaneous enzymes | 4.70E-06 | 8.12E-04 |
| | 26.1 | miscellaneous enzymes, cytochrome P450 | 6.81E-07 | 1.18E-04 |
| | 26.16 | miscellaneous enzymes, myrosinases-lectin-jacalin | 3.11E-05 | 5.38E-03 |
| | 35 | not assigned | 7.03E-05 | 1.22E-02 |
| | 35.3 | not assigned, new | 8.67E-07 | 1.50E-04 |
| | 16.8.4 | secondary metabolism, flavonoids, flavonols | 2.09E-04 | 3.62E-02 |
| | 30.2.24 | signaling, receptor kinases, S-locus glycoprotein like | 1.42E-04 | 2.45E-02 |
| | 34.13 | transport, peptides and oligopeptides | 1.84E-04 | 3.18E-02 |

| Cluster | Bin number | Bin name | p value | Adjusted p value |
|---|---|---|---|---|
| 5 | 26.4 | miscellaneous enzymes, beta 1,3 glucan hydrolases | 2.60E-08 | 5.00E-06 |
| | 3.2.2 | minor CHO metabolism, trehalose, TPP | 1.60E-04 | 3.07E-02 |
| 6 | 33 | development | 7.59E-06 | 6.68E-04 |
| | 33.1 | development, storage proteins | 3.00E-10 | 2.64E-08 |
| | 17.7 | hormone metabolism, jasmonate | 2.96E-04 | 2.61E-02 |
| | 17.7.1 | hormone metabolism, jasmonate, synthesis/degradation | 2.75E-04 | 2.42E-02 |
| | 26.8 | miscellaneous enzymes, nitrilases, nitrile lyases, berberine bridge enzymes, reticuline oxidases, troponine reductases | 1.79E-11 | 1.57E-09 |
| | 35 | not assigned | 2.97E-07 | 2.62E-05 |
| | 20.2.99 | stress, abiotic, unspecified | 3.04E-04 | 2.68E-02 |
| | 20.1.7 | stress, biotic, PR-proteins | 7.22E-06 | 6.36E-04 |
| | 20.1.7.6 | stress, biotic, PR-proteins, proteinase inhibitors | 8.05E-05 | 7.09E-03 |

Table S5. Enrichment of GO terms in the 6 clusters shown on Figure 3. P values adjusted with Bonferroni.

| Cluster | GO accession number | Term Type | Term | p value | Adjusted p value |
|---|---|---|---|---|---|
| 1 | GO:0003824 | F | catalytic activity | 8E-05 | 0.001 |
| | GO:0030312 | C | external encapsulating structure | 9E-09 | 1E-07 |
| | GO:0005576 | C | extracellular region | 3E-12 | 4E-11 |
| | GO:0044421 | C | extracellular region part | 2E-07 | 2E-06 |
| | GO:0005615 | C | extracellular space | 9E-08 | 1E-06 |
| | GO:0016787 | F | hydrolase activity | 0.001 | 0.017 |
| | GO:0016491 | F | oxidoreductase activity | 0.003 | 0.03 |
| | GO:0050896 | P | response to stimulus | 2E-04 | 0.003 |
| 2 | GO:0003824 | F | catalytic activity | 0.004 | 0.034 |
| 3 | NS | | | | |
| 4 | GO:0016301 | F | kinase activity | 9E-04 | 0.015 |
| | GO:0016020 | C | membrane | 2E-05 | 0.0003 |
| | GO:0060089 | F | molecular transducer activity | 0.002 | 0.043 |
| | GO:0016491 | F | oxidoreductase activity | 7E-05 | 0.0013 |
| | GO:0004872 | F | receptor activity | 4E-04 | 0.0067 |
| | GO:0004871 | F | signal transducer activity | 0.002 | 0.043 |
| | GO:0016772 | F | transferase activity, transferring phosphorus-containing groups | 9E-04 | 0.015 |
| 5 | GO:0003824 | F | catalytic activity | 0.001 | 0.025 |
| | GO:0005576 | C | extracellular region | 1E-07 | 2E-06 |
| | GO:0005615 | C | extracellular space | 8E-06 | 0.0001 |
| | GO:0044421 | C | extracellular region part | 2E-05 | 0.0002 |
| 6 | GO:0005576 | C | extracellular region | 1E-03 | 0.0077 |

Key: P, biological process; F, molecular function and C, cellular compartment, NS, not significant.
